# Perception of illness among patients with heart failure is related to their general health independently of their mood and functional capacity

**DOI:** 10.1186/s41687-019-0142-1

**Published:** 2019-08-16

**Authors:** Anners Lerdal, Dag Hofoss, Caryl L. Gay, May Solveig Fagermoen

**Affiliations:** 1 Department of Nursing Science, Institute of Health and Society, Faculty of Medicine, University of Oslo, P.O. Box. 1130, N-0318 Oslo, Blindern Norway; 2 Department for Patient Safety and Research, Lovisenberg Diaconal Hospital, P.O. Box 04970, Nydalen, N-0440 Oslo, Norway; 3 Lovisenberg Diaconal University College, Lovisenberggata 15b, N-0456 Oslo, Norway; 4 Department of Family Health Care Nursing, University of California, San Francisco, 2 Koret Way, San Francisco, CA 94143 USA; 5 Department of Health Management and Health Economics, Faculty of Medicine, Institute of Health and Society, University of Oslo, P.O. Box. 1130, Blindern, N-0318 Oslo, Norway

**Keywords:** Illness perception, Heart failure, Depression, Anxiety, Quality of life, Functioning

## Abstract

**Purpose:**

To explore the relationship between illness perceptions and self-reported general health of patients with chronic heart disease, using some core elements from the Common Sense Model.

**Methods:**

Patients with heart failure (New York Heart Association [NYHA] Functional Class I-III) from five outpatient clinics in Eastern Norway were invited to participate in this cross-sectional study. Two research nurses collected socio-demographic data (age, sex, education and work status) and standardized questionnaires in structured interviews. Patients’ self-reported general health was measured with the Euro-Qual Visual Analogue Scale (EQ-VAS), illness perceptions were measured with the 8-item Brief Illness Perception Questionnaire (BIPQ), and mood was assessed using the Hospital Anxiety and Depression Scale.

**Results:**

Among the 220 patients who were recruited into this study (98% response rate), the mean age was 67.5 years (SD ± 12.5), and 65.9% were men. Patients were classified as NYHA Class I (8.7% with no activity limitations), Class II (47.6% with slight limitations), or Class III (43.8% with marked limitations). Mean EQ-VAS score was 58.8 (SD ± 21.0). Three of the eight perception of illness items (consequences, personal control and identity) were associated with the patients’ general health rating, controlling for their NYHA Class, mood and other BIPQ items.

**Conclusions:**

Our findings suggest that patients’ perceptions of their illness have an independent and substantial relationship to the self-rated general health of patients with chronic heart failure. Peoples’ illness perceptions are beliefs that have been shown to be modifiable in clinical interventions. Thus, targeted interventions aimed to modify these, such as patient education courses, ought to be developed and tested, as they may be helpful for improving perceived health status.

## Introduction

Heart failure (HF) is a severe condition affecting many people throughout the world and is the most frequent reason for hospitalization in the aging population [1]. HF is a chronic disease and is particularly common in high-income countries [2]. In Norway, more than 100,000 people suffer from HF, one-third of whom are hospitalized each year [3]. Moreover, the prevalence of HF is expected to increase as the population continues to age [4].

Chronic heart failure (CHF) is clinically defined as a syndrome due to abnormality in the patient’s cardiac structure or function with typical signs and symptoms due to sodium and water retention. The patient’s experience involves symptoms such as dyspnea, ankle edema and fatigue, which often result in reduced quality of life [5, 6]. Studies have reported high levels of depression and anxiety in patients with HF [7, 8]. A meta-study on HF and depression concluded that depression as a comorbid illness in heart failure has significant implications for the patient, both in terms of quality of life and health outcomes [8].

Living with chronic illness involves coping with symptoms and distress to maintain quality of life. The person’s perceptions of their illness form the cognitive basis for his or her adaptive coping responses [9, 10]. The Common Sense Model (CSM) of illness describes ways patients develop and organize their beliefs about their illness in order to make sense of and cope with their situation [9]. Illness representation is affected by many elements, e.g. age, sex, employment, medical information and advice, symptoms and problems in living with the illness. Information about the disease is processed selectively in the patient’s own mind and creates his or her own understanding of the illness.

**Fig. 1:**
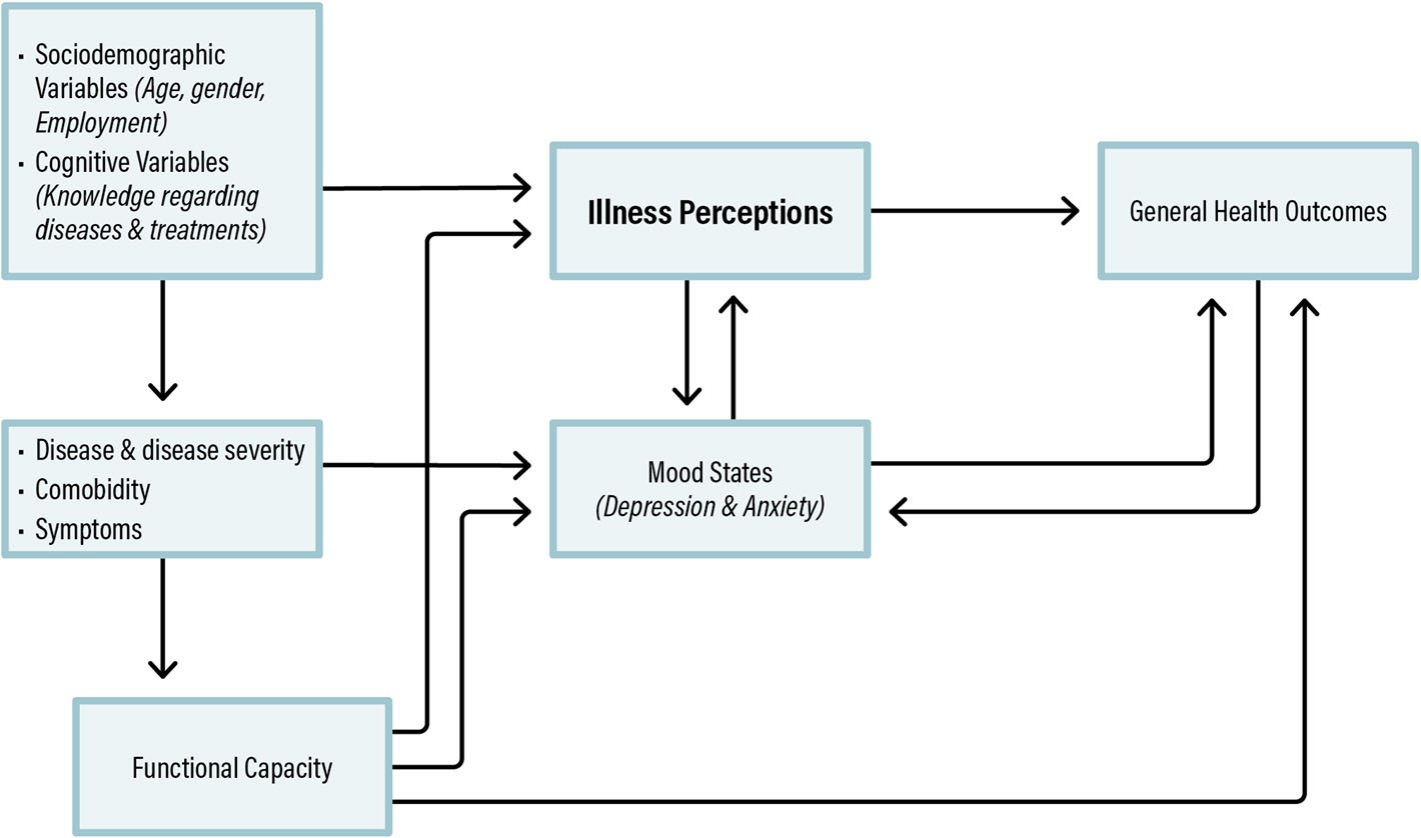
Theoretical framework for the study based on the Common Sense Model of illness

The CSM is the general theoretical orientation with which we framed this study by adopting its general premise that illness representations affect health outcomes [11]. Thus, the focus in our study was on examining the role of functional capacity inherent in chronic illness as another dimension in this process mode, and on the relationships among illness perceptions, functional capacity, and perceived health outcomes in patients with heart failure (see Fig. 1). Since emotional mood states, especially depression and anxiety, have been found to be major issues in patients with heart failure, we also included mood states as an additional aspect in this model.

Since theoretically, patients’ illness perceptions are believed to be influenced by information delivered in patient education, it is important to determine whether there is a relationship between illness perceptions and self-reported health, controlling for the mood states of anxiety and depression and other relevant factors [12]. Based on prior findings, there is a growing interest in exploring the relationship between patients’ illness beliefs and their self-reported health and quality of life.

### Aims

1. To explore the relationships between illness perceptions, sex and functional capacity of patients with chronic heart failure.
2. To explore the relationships between patients’ perceptions of both their illness and general health, accounting for other relevant factors, such as socio-demographic indices (sex, age, education, and work status), clinical characteristics, and the mood states of anxiety and depression.

## Methods

### Procedure and participants

Patients were recruited by nurses at heart failure (HF) outpatient clinics at six hospitals in Eastern Norway. The inclusion criteria were: (1) diagnosis of chronic HF with a preserved or non-preserved ejection fraction; (2) previous evaluation of HF, exposure to optimal medical therapy, and use of medication for at least 1 month prior to the study; (3) the ability to read and speak Norwegian.

Patients were excluded if they had: (1) observed cognitive impairment; (2) co-morbid life-threatening illness such as cancer or chronic renal failure; (3) history of myocardial infarction within 3 months of the study; or (4) history of a cerebral vascular accident 3 months prior to the study or any major sequelae of HF. Eligible patients received written information about the study and an invitation to participate. The nurse researchers conducted a structured interview to complete a data collection questionnaire while the patients followed along with their own copy of the questionnaire.

### Measures

**Socio-demographic indices** included sex, age (in years), education level (lower than high school, high school or university), and work status (paid work or not).

**Functional capacity** was measured by the patient’s physician according to the New York Heart Association (NYHA) classification of heart failure as mild (I), moderate (II), severe (III) or very severe (IV) [13]. In this study, patients diagnosed with NYHA class I-III were approached. NYHA class was collected from the patients’ medical record.

**Self-reported general health** was assessed using the Euro-Qol measure EQ-VAS [14]. Participants were asked to mark how good or bad their health is today on a visual analog scale (VAS) ranging from 0 (worst imaginable health) to 100 (best imaginable health).

**Mood states** were assessed using the Hospital Anxiety and Depression Scale (HADS) [15]. This 14-item instrument measures current symptoms of anxiety and depression and yields a subscale score for each. The items are rated on a four-point scale, 0 (Not at all) to 3 (Very much). The anxiety and depression subscale scores are categorized as normal (< 7), borderline (8–11), or probable/case of anxiety or depression morbidity (> 11). The HADS is widely used and has been shown to have good reliability and validity when used in Norwegian setting [16, 17].

**Illness perceptions** were assessed with the Brief Illness Perception Questionnaire (BIPQ) [12, 18]. The BIPQ consists of the following eight items: 1) *Consequences* - How much does your illness affect your life?; 2) *Timeline* - How long do you think your illness will continue?; 3) *Personal control* - How much control do you feel you have over your illness?; 4) *Treatment control* - How much do you think your treatment can help your illness?; 5) *Identity -* How much do you experience symptoms from your illness?; 6) *Illness concern* - How concerned are you about your illness?; 7) *Coherence* - How well do you feel you understand your illness?; and 8) *Emotional representation* - How much does your illness affect you emotionally? The items are rated on a 0–10 scale, where higher scores indicated more of the concept being measured.

### Ethics

The study was approved by the Regional Committee on Medical Ethics in Norway (REC S-08288a-2008/8618) and conforms to the Helsinki Declaration. The participants signed an informed consent form prior to participation in the study.

### Data analyses

Group differences in categorical variables were tested by chi-squared tests, except Fisher’s exact test was applied instead if more than 20% of the expected frequencies were less than 5. Group differences in means scores were evaluated by independent samples t-tests and one-way analysis of variance. Where more than two group averages were compared, *p*-values were adjusted with Bonferroni’s or Tamhane’s T2 corrections, according to whether group variances could be considered equal or not (group variance equalities were tested by Levene’s test). Relationships of socio-demographic background variables (age, sex, education, and employment status), functional capacity (NYHA class), mood (anxiety and depression), and illness perception (the eight BIPQ-components) to self-reported general health (EQ VAS 0–100) were analyzed by multiple linear regression. In each of the four models analyzed, all variables (4, 6, 10, and 18 for the four models, respectively) were entered simultaneously. Differences and regression coefficients whose *p*-values did not exceed .05 were considered significant.

## Results

The sample’s socio-demographic and clinical characteristics are described in Table 1. A total of 220 subjects participated in the study (98% response rate): 156 men (71%) and 64 women (29%). The mean age was 67 years (69.8 years for women and 66.6 years for men) and ranged from 34 to 103 years; 153 (71%) had completed high school, 131 (59%) were married, 122 (55%) were retired, and 82 (37%) were living alone.

**Table 1:** Demographic, clinical characteristics, self-reported general health and illness perceptions by sex

Characteristics	Total sample *N* = 220	Male *n* = 156	Female *n* = 64	*P*-value
Age, mean (SD)	67.5 (12.5)	66.6 (12.2)	69.8 (13.1)	.08
Education level, N (%)	*N* = 215	*n* = 152	*n* = 63	.56
< High school	71 (33.0)	48 (31.6)	23 (36.5)	
High school	82 (38.1)	57 (37.5)	25 (39.7)	
≥ University	62 (28.8)	47 (30.9)	15 (23.8)	
Work status, N (%)	*N* = 216	*n* = 152	*n* = 64	.46
Working	33 (15.3)	25 (16.3)	8 (12.7)	
Not working	183 (84.7)	128 (83.7)	55 (87.3)	
Functional capacity, N (%)	*N* = 208	*n* = 146	*n* = 62	**.006**
NYHA Class I	18 (8.7)	16 (11.0)	2 (3.2)	
NYHA Class II	99 (47.6)	76 (52.1)	23 (37.1)	
NYHA Class III	91 (43.8)	54 (37.0)	37 (59.7)	
HADS Anxiety, mean (SD)	4.7 (4.0)	4.3 (3.3)	5.7 (3.8)	**.019**
HADS Anxiety, N (%)	*N* = 219	*n* = 156	*n* = 63	.12^a^
Normal 0–7	168 (76.7)	125 (80.1)	43 (68.3)	
Borderline 8–10	35 (16.0)	20 (12.8)	15 (23.8)	
Case of morbidity ≥11	16 (7.3)	11 (7.1)	5 (7.9)	
HADS Depression, mean (SD)	4.8 (3.5)	4.8 (3.7)	5.0 (3.3)	.79
HADS Depression, N (%)	*N* = 220	*n* = 156	*n* = 64	.94
Normal 0–7	177 (80.5)	126 (80.8)	51 (79.7)	
Borderline 8–10	25 (11.4)	17 (10.9)	8 (12.5)	
Case of morbidity ≥11	18 (8.2)	13 (8.3)	5 (7.8)	
Self-reported general health	*N* = 213	*n* = 150	*n* = 63	
EQ-VAS, mean (SD)	53.81 (20.96)	54.15 (21.51)	52.98 (1.77)	.72
Illness perceptions, mean (SD)	*N* = 220	*n* = 156	*n* = 64	
Consequences	5.77 (2.5)	5.69 (2.5)	5.95 (2.5)	.49
Timeline	9.07 (1.9)	8.98 (1.9)	9.29 (1.9)	.28
Personal control	5.94 (2.3)	5.79 (2.3)	6.30 (2.1)	.14
Treatment control	7.84 (2.1)	7.77 (2.1)	8.02 (1.9)	.42
Identity	5.45 (2.7)	5.15 (2.7)	6.17 (2.5)	**.01**
Illness concern	4.26 (3.1)	4.03 (3.1)	4.83 (3.0)	.08
Coherence	7.11 (2.2)	7.26 (2.1)	6.77 (2.5)	.17
Emotional representation	4.62 (3.0)	4.35 (3.1)	5.30 (2.8)	**.03**

^a^Fishers exact test due to expected count less than 5. Note. Bold *p*-values are < .05

Patients with NYHA Class I-III who attended any of the outpatient clinics were included in the study. Female patients were more likely than men to be classified as NYHA class III. There were no sex differences with respect to age, level of education, work status, mood (anxiety or depression) or self-reported general health. Except for higher scores on *illness identity* and *emotional representation* among women than among men, our analyses did not reveal any sex difference in illness perceptions (Table 1).

Mean illness perception scores among patients differed by level of functional capacity (i.e. NYHA classes). Sub-analyses indicated several differences in the eight illness perception items in relation to NYHA class, except for *timeline* and *coherence* as shown in Table 2. In general, worse functional capacity was associated with more negative illness perceptions.

**Table 2:** Illness perception mean scores (standard deviation) by functional capacity (*N* = 208)

*Illness perceptions, Mean (SD)*	NYHA Class I (*n* = 18)	NYHA Class II (*n* = 99)	NYHA Class III (*n* = 91)	Pairwise *p*-values
Consequences How much does your illness affect your life?	3.67 (2.3)	5.43 (2.4)	6.49 (2.2)	**1 vs 2:** ***p*** **= .01** **1 vs 3:** ***p*** **< .001** **2 vs 3:** ***p*** **= .01**
Timeline How long do you think your illness will continue?	8.56 (1.9)	8.93 (2.1)	9.34 (1.6)	1 vs 2: *p* = .84 1 vs 2: *p* = .84 2 vs 3: *p* = .33
Personal control How much control do you feel you have over your illness?	7.39 (1.7)	5.96 (2.2)	5.71 (2.3)	**1 vs 2:** ***p*** **= .03** **1 vs 3:** ***p*** **= .01** 2 vs 3: *p* > .99
Treatment control How much do you think your treatment can help your illness?	8.94 (1.1)	7.91 (1.8)	7.57 (2.3)	**1 vs 2:** ***p*** **= .01** **1 vs 3:** ***p*** **= .001** 2 vs 3: *p* = .60
Identity How much do you experience symptoms from your illness?	2.72 (2.2)	5.01 (2.5)	6.45 (2.3)	**1 vs 2:** ***p*** **= .001** **1 vs 3:** ***p*** **< .001** **2 vs 3:** ***p*** **< .001**
Illness concern How concerned are you about your illness?	2.78 (2.1)	3.82 (2.8)	4.80 (3.3)	1 vs 2: *p* = .53 **1 vs 3:** ***p*** **= .03** 2 vs 3: *p* = .07
Coherence How well do you feel you understand your illness?	6.50 (2.3)	7.01 (2.0)	7.21 (2.3)	1 vs 2: *p* > .99 1 vs 3: *p* = .64 2 vs 3: *p* > .99
Emotional representation How much does your illness affect you emotionally?	2.33 (1.8)	4.56 (3.1)	5.09 (2.8)	**1 vs 2:** ***p*** **= .01** **1 vs 3:** ***p*** **= .001** 2 vs 3: *p* = .62

Note: Anova tests were used for all comparisons. Where the Levene test indicated homogeneity of variance could be assumed, Bonferroni’s adjustment for multiple comparisons was applied; where homogeneity of variance could not be assumed, Tamhane’s T2 adjustment was applied. Bold *p*-values are < .05

The relationships between the patients’ perception of their general health, socio-demographic and clinical characteristics, mood, and perception of illness are shown in Table 3. When we assessed the relationship between perceptions of general health and socio-demographic variables (Model 1), having paid work was related to better general health, controlling for the effects of age, sex and level of education. This relationship remained significant when the patients’ functional capacity was included in the regression model (Model 2). In this model, better functional capacity (lower NYHA class) was also related to perceptions of better general health. When mood was added to the model (Model 3), only better functional capacity and lower (normal) scores on depression were related to better self-reported general health. The findings were similar when mood was evaluated as continuous HADS scores, rather than categories (results not shown). In the final model (Model 4), all eight illness perception variables were introduced simultaneously. This multivariate model showed that lower illness *consequences*, higher *personal control*, and lower illness *identity* were associated with better self-reported general health, controlling for socio-demographic factors, functional capacity and mood states. This final model explained 44.1% of the variance in self-reported general health. When the illness perception variables were included in the regression model, the explained variance increased by another 14.4%, i.e. by a factor of 1.5.

**Table 3:** Regression analysis of self-reported general health (EQ VAS 0–100) on sociodemographic variables (Model 1), and functional capacity (Model 2), and mood (Model 3), and illness perceptions (Model 4)

Predictors	Model 1 (*n* = 192)	Model 2 (*n* = 181)	Model 3 (*n* = 180)	Model 4 (*n* = 177)
	β (p)	β (p)	β (p)	β (p)
Step 1: Socio-demographics				
Age (years)	.02 (.78)	.07 (.38)	.01 (.95)	−.08 (.29)
Sex (reference: male)	−.04 (.62)	.03 (.72)	.04 (.57)	.02 (.76)
Education (reference: < 12 years)	.09 (.26)	.06 (.39)	.04 (.54)	.07 (.23)
Work (reference: not working)	.26 **(.003)**	.16 **(.050)**	.14 (.07)	.07 (.29)
Step 2: Functional capacity				
NYHA Class II (reference: NYHA Class I)		.41 **(<.001)**	.33 **(<.001)**	.14 (.052)
NYHA Class III (reference: NYHA Class I)		.14 (.068)	.11 (.134)	−.02 (.73
Step 3: Mood states				
Anxiety Borderline (reference: normal 0–7)			−.07 (.33)	−.02 (.83)
Anxiety Morbidity (reference: normal 0–7)			−.01 (.93)	.02 (.80)
Depression Borderline (reference: normal 0–7)			−.16 **(.02)**	−.12 (.08)
Depression Morbidity (reference: normal 0–7)			−.34 **(<.001)**	−.27 **(<.001)**
Step 3: Illness perceptions (BIPQ)				
Consequences				−.30 **(<.001)**
Timeline				.04 (.49)
Personal control				.17 **(.012)**
Treatment control				−.04 (.56)
Identity				−.21 **(.008)**
Concern				−.05 (.57)
Coherence				−.02 (.76)
Emotional representation				.10 (.26)
Model-specific explained variance (R^2^_adj_)	.055	.172	.297	.441

Note. Bold *p*-values are < .05

## Discussion

The main finding of this study of patients with heart failure was that their cognitive illness perceptions, i.e. *consequences*, *personal control* and *identity*, were significantly associated with their self-reported general health, as hypothesized based on our conceptual model (Fig. 1). The relationships between these illness perceptions and self-rated general health were independent of the patients’ functional capacity, and mood states. Controlling for mood symptoms is important, as previous studies have shown a relationship between mood and illness perceptions [19], which could confound our observed association between illness perceptions and perceived general health.

The role of illness perceptions has received relatively little attention in cardiovascular disease compared to the role of depression, which has been studied for decades [20]. Nonetheless, our findings are consistent with what other studies have found: illness perceptions have substantial associations with self-reported health [21]. However, to our knowledge, this is the first cross-sectional study to show this relationship between illness perceptions and self-rated general health after controlling for socio-demographic variables, functional capacity, depression and anxiety. A longitudinal study showed that lower scores on illness *identity* at baseline predicted better quality of life in patients with congenital heart disease after 1 year [19]. However, that study did not control for the patients’ mood.

The independent variables in our analytical model explained a relatively high proportion of the participants’ self-reported health. This could indicate that, from the patient’s perspective, illness perceptions, functioning, and mood states, such as anxiety and depression, may be important factors to address in recovery and rehabilitation for patients with heart diseases. Illness perceptions represent the person’s thoughts and beliefs about his or her illness. A randomized controlled study in patients with myocardial infarction showed that illness perception can be modified through a patient education course or similar intervention [22]. Thus, evidence has shown that personal beliefs about illness can be modified by patient education.

### Study strengths and limitations

Strengths of this study include the integration of a theoretical approach based on the core concepts of the CSM and the identification of the relationship between illness perceptions and self-reported health after controlling for mood in the analysis. A limitation of this cross-sectional study is that we cannot determine whether the observed relationship is causal in nature. Although we controlled for physical capacity (NYHA class) in the regression models, it cannot be ruled out that the patients’ health status influences their illness perceptions rather than the reverse or that their association is not causal at all. Intervention studies are needed to determine whether modifying patients’ illness perceptions can improve their health outcomes. In addition, this study used NYHA class to assess functional capacity. Although this is a well-established classification system, it is a rather crude measure and may not have been able to distinguish more subtle differences in disease severity. Future studies should consider additional clinical measures to better account for the role of patients’ current symptoms and health status. The patients’ ethnicity and religious beliefs may also be associated with their illness perception. However, as we have not measured these variables, we were not able to explore the role of these factors in this study.

## Conclusion

Our findings suggest that patients’ perceptions of their illness, i.e. their lay beliefs about its consequences, their personal control over it and its identity in their lives, have an independent and substantial relationship to perceived general health among patients with chronic heart failure. These relationships remained significant even after controlling for the patients’ mood states of anxiety and depression and their functional capacity. Peoples’ illness perceptions are beliefs that have been shown to be modifiable in clinical interventions. Thus, targeted interventions aimed to modify these factors (e.g., through patient education courses) ought to be developed and tested to determine whether they might be helpful for improving patients’ perceived health status.

## Data Availability

Please contact author for data requests.

## Abbreviations

BIPQ: Brief Illness Perception Questionnaire
CHF: Chronic heart failure
CSM: Common Sense Model
EQ-VAS: Euro-Qol Visual Analogue Scale
HADS: Hospital Anxiety and Depression Scale
HF: Heart failure
NYHA class: New York Heart Association classification of heart failure
REC: Regional Committee on Medical Ethics
